# Isolation and genetic analysis of an *Enterococcus gallinarum* strain with high GABA-producing capacity from the murine intestine

**DOI:** 10.1016/j.bbrep.2026.102603

**Published:** 2026-04-24

**Authors:** Mion Ikegami, Miyu Yamashita, Ren Kadowaki, Gaku Harata, Kenji Miyazawa, Yusuke Nakanishi, Kyoko Takahashi

**Affiliations:** a Nihon University Graduate School of Bioresource Sciences, Kanagawa, 252-0880, Japan; b College of Bioresource Sciences, Nihon University, Kanagawa, 252-0880, Japan; c Technical Research Laboratory, Takanashi Milk Products Co., Ltd, Kanagawa, 241-0023, Japan

**Keywords:** Gut microbiota, γ-Aminobutyric acid, *Enterococcus gallinarum*, *gad* cluster

## Abstract

Some intestinal bacteria possess the glutamate decarboxylase (*gad*) gene and can produce γ-aminobutyric acid (GABA), a major inhibitory neurotransmitter in mammals, from glutamate. The physiological role of gut-microbiota-derived GABA has attracted attention because of the relationships identified between gut microbiota and psychiatric and neurological disorders. We screened for GABA-producing or GABA-consuming bacteria in the murine intestine using selective media targeting two groups of bacteria that were reduced or increased in children with autism spectrum disorder: Lactobacillales and Clostridiales. As a result, an *Enterococcus gallinarum* strain belonging to Lactobacillales was identified as a high GABA producer. The isolated strain possesses the *gad* cluster and produced larger amounts of GABA from glutamate than the *Levilactobacillus brevis* strain, a well-known GABA producer. This study identified a novel gut-resident bacterium with a high GABA production capacity as a potential source of intestinal GABA.

## Highlights

• An *Enterococcus gallinarum* strain was isolated from the murine intestine.
• This strain produced high levels of GABA from glutamate.
• Whole-genome sequencing revealed the presence of a *gad* gene cluster.
• The cluster contained *gad*C, *gad*, and a gene encoding helix-turn-helix protein.

## 1 Introduction

The gut microbiota that inhabits the intestinal tract has emerged as a key player in maintaining host health. Its disturbances correlate with various diseases such as allergies, cancer, and diabetes. Recently, the influence of gut microbiota on neurological and psychiatric disorders has attracted increasing attention from the perspective of the gut-brain axis.

Autism spectrum disorder (ASD) is one of the most studied psychiatric disorders in relation to gut microbiota, as the composition of gut microbiota is imbalanced in both patients with ASD and mouse models of ASD. Reduced levels of *Bifidobacterium* and *Lactobacillus* and an increase in *Clostridium* in the intestine are associated with abnormal behaviors, such as anxiety and hyperactivity, in children with ASD [1]. Some *Lactobacillus* strains, including *L*. *reuteri* and *L. plantarum,* reduce social deficits in ASD mouse models [2,3]. Conversely, the abundances of *Clostridium perfringens*, *Clostridium histolyticum* and several other *Clostridium* species are higher in the feces of children with ASD compared to healthy controls, indicating that *Clostridium* species are risk factors for ASD [[4], [5], [6]]. Therefore, intervention in the gut microbiota via administrating probiotics is expected to alleviate the symptoms of ASD by affecting not only the intestinal functions but also the brain via complex neuronal and immunological cascades. Accumulating evidence suggests that gut-microbe-derived metabolites primarily mediate effects on neuronal and psychiatric disorders. These metabolites include various neurotransmitters and neuromodulators, and we focused on GABA, a major inhibitory neurotransmitter in mammals. GABA dysfunction is associated with abnormal behaviors related to ASD as well as schizophrenia because GABA maintains the ratio of excitatory to inhibitory cortical activity, which is crucial for neurological balance [[7], [8], [9]].

GABA is synthesized from glutamate by glutamate decarboxylase (GAD). Some intestinal bacteria, such as *Levilactobacillus brevis* and *Bifidobacterium dentium*, possess the *gad* gene and can produce GABA [[10], [11], [12], [13], [14], [15]]. ASD or depression and glutamate/GABA metabolism by the gut microbiota were correlated in metagenomic studies in humans and mice [16,17]. Furthermore, the fecal GABA content was lower in children with ASD than in the control group, accompanied by differences in the composition of gut microbiota, suggesting a physiological role of intestinal GABA derived from the microbiota [18]. A recent report shows that infants with low-likelihood ASD have higher amounts of fecal GABA than infants with elevated-likelihood ASD at 5 months, which positively and negatively corelates with the abundances of *Bifidobacterium* and *Lactobacillus* species and *Clostridium* species in the gut microbiota, respectively [19]. We consistently found that administration of *Bifidobacterium bifidum* TMC3115, a probiotic candidate without *gad*, elevated the GABA levels in the gut and reduced anxiety-like behavior in mice, with an increase and a decrease in the abundance of Lactobacillales and Clostridiales species, respectively [20].

In this study, we screened bacteria from the murine intestine that produce or consume GABA within the orders Lactobacillales and Clostridialales, the abundances of which strongly correlate with altered neuronal activity or abnormal behavior in ASD. We identified *Enterococcus gallinarum,* a bacterium of the order Lactobacillales, as a high GABA producer, suggesting its contribution to maintaining the intestinal GABA concentration.

## 2 Materials and methods

### 2.1 Isolation of bacteria from murine intestine using selection media for Lactobacillales and Clostridiales

The frozen cecum contents of 10-week-old female BALB/c mice were suspended in saline at a concentration of 0.1 g/mL. The contents were serially diluted and spread on Man–Rogosa–Sharpe (MRS) agar medium (BD Biosciences, Franklin Lakes, NJ, USA) to isolate Lactobacillales bacteria. To isolate Clostridiales bacteria, the cecum contents were heat-treated at 70 °C for 20 min, serially diluted, and spread on Clostridia Count Agar Medium (Shimadzu Diagnostics, Tokyo, Japan). After 24 h of anaerobic incubation at 37 °C, characteristic colonies were randomly selected for 16S rRNA gene capillary sequencing analysis. Animal experiments were approved by the Nihon University Animal Care and Use Committee and were conducted in accordance with their guidelines. Mice were purchased from CELA Japen (Tokyo, Japan) and the cecal contents obtained from the experiment with approval number AP21BRS057 were used in this study.

### 2.2 16S rRNA sequencing

DNA was extracted from each colony using PrepMan Ultra Sample Preparation Reagent (Applied Biosystems, Waltham, MA, USA). The 16S rRNA gene region was amplified and sequenced using a MicroSeq 500 16S Bacterial Identification Kit (Applied Biosystems) according to the instructions provided by the manufacturer.

### 2.3 Whole-genome sequencing

Genomic DNA was extracted and purified using a NucleoSpin DNA Stool kit (MACHEREY-NAGEL GmbH & Co., KG, Düren, Germany) following the manufacturer's instructions. The library was prepared using a Nextera DNA Flex Library Prep Kit (Illumina, San Diego, USA). Sequencing was performed using MiSeq (Illumina) with 300 bp paired-end reads. The sequencing reads were quality-controlled and cleaned using fastp v.0.25.0, with default settings. The reads were then assembled using Platanus_B v.1.3.2 [21,22]. The assembled sequences were annotated using the DNA Data Bank of Japan Fast Annotation and Submission Tool (DFAST) v.1.6.0 [23]. The completeness and contamination rates of the genome were assessed using CheckM2 v.1.0.2 [24].

### 2.4 Preparation of bacterial culture supernatants

Single bacterial colonies were inoculated into 5 mL of medium and cultured anaerobically at 37 °C for 30 h. Anaerobic cultivation was performed using an AnaeroPack system (Mitsubishi Gas Chemical, Tokyo, Japan) in a sealed jar. The internal environment was maintained under anaerobic conditions (<0.1% O_2_ and >15% CO_2_). Then, 50 μL of each culture was inoculated into 5 mL of new medium supplemented with either 1 % l-glutamate or GABA. MRS and Clostridium count media were used for culturing Lactobacillales and Clostridiales, respectively. After culturing for 24 h at 37 °C under anaerobic conditions, the culture supernatants were collected by centrifuging at 6000×*g* for 10 min at 4 °C, filtered through 0.20 μm filters, and stored at −80 °C until use. Control media without the bacterial inoculum were prepared similarly. *Levilactobacillus brevis* NBRC 12005 was obtained from the National Institute of Technology and Evaluation (Tokyo, Japan) and was used as the control.

### 2.5 Quantification of GABA

The GABA concentrations in the culture supernatants were quantified using a GABA Assay Kit (Enzyme Sensor, Ibaraki, Japan) according to the manufacturer's instructions, with one modification: the experimental scale was reduced to 1/5 of the original volume to enable measurement using a 96-well plate. The GABA concentrations (w/v) in the culture supernatants were determined using a calibration curve generated with serially diluted GABA standards.

### 2.6 Quantification of glutamate

The glutamate concentrations in the culture supernatants were quantified using a Glutamate Assay Kit (Abcam, Cambridge, U.K.), according to the instructions provided by the manufacturer. A calibration curve was prepared using serially diluted glutamate standards.

### 2.7 Visualization of gene clusters

The genomic region surrounding *gad* and the syntenic context were visualized using Clinkerv.0.0.31 [25].

### 2.8 Construction of a phylogenetic tree

Sequences were aligned using the MUSCLE method implemented with MEGA X software [26]. Phylogenetic trees were constructed using the maximum-likelihood method with 1000 bootstrap replicates. The resulting ML phylogenetic trees were visualized using iTOL [27]. Bootstrap values greater than 80%, based on 1000 replicates, are shown at the branch nodes.

## 3 Results

### 3.1 Isolation of Lactobacillales and Clostridiales bacteria from murine intestine

First, we attempted to isolate Lactobacillales or Clostridiales bacteria from the murine cecum contents using the selective media for their bacterial orders. Two isolates grown on MRS medium were thought to be *Lactobacillus murinus* and *Enterococcus faecium* based on the 16S rRNA gene sequencing. However, the subsequent whole-genome sequence analysis revealed that both isolates were *Enterococcus gallinarum.* The genomic sequences of the two strains more than 99 % matched, indicating that these were the same strains. Nucleotide sequences were deposited at DDBJ under accession numbers BAAIJE010000001-BAAIJE010000121 and BAAIJF010000001-BAAIJF010000349 (https://getentry.ddbj.nig.ac.jp). Six isolates grown on clostridia count media were identified as *Clostridium aerotolerans*, *Clostridium diolis* (two strains), *Clostridium argentinense*, *Clostridium magnum*, and *Asaccharospora irregularis* by analyzing the 16S rRNA gene sequences. The obtained isolates are summarized in Table 1.

**Table 1:** List of isolated bacteria.

Selective media	Isolated bacteria
MRS agar medium	*Enterococcus gallinarum*^*a*^
Clostridia count agar medium	*Clostridium aerotolerans*^*b*^

	*Clostridium diolis* (2 strains)^b^
	*Clostridium argentinense*^*b*^
	*Clostridium magnum*^*b*^
	*Asaccharospora irregularis*^*b*^

Bacteria were isolated from murine cecum contents using selective media and identified by 16S rRNA gene (a) or whole-genome (b) sequencing.

### 3.2 GABA production and consumption abilities of isolated intestinal bacteria

**Fig. 1:**
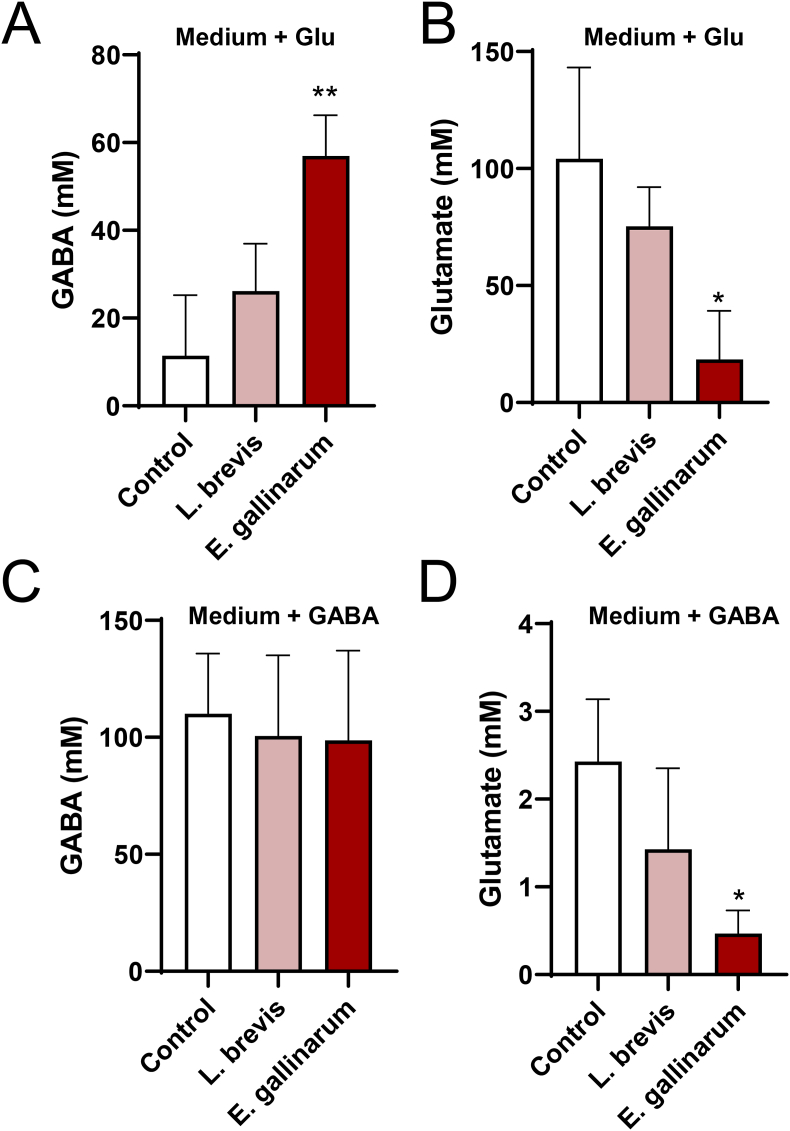
**GABA production and consumption ability of isolated Lactobacillales.** The isolated *E. gallinarum* strain and *L. brevis* NBRC 12005 were cultured in media supplemented with glutamate (A, B) or GABA (C, D) to determine the change in GABA (A, C) and glutamate (B, D) concentrations in the culture supernatants. Data are presented as mean ± SD of three independent experiments. *P < 0.05 and **P < 0.01 vs. control without bacteria (one-way ANOVA with Dunnett's test).

**Fig. 2:**
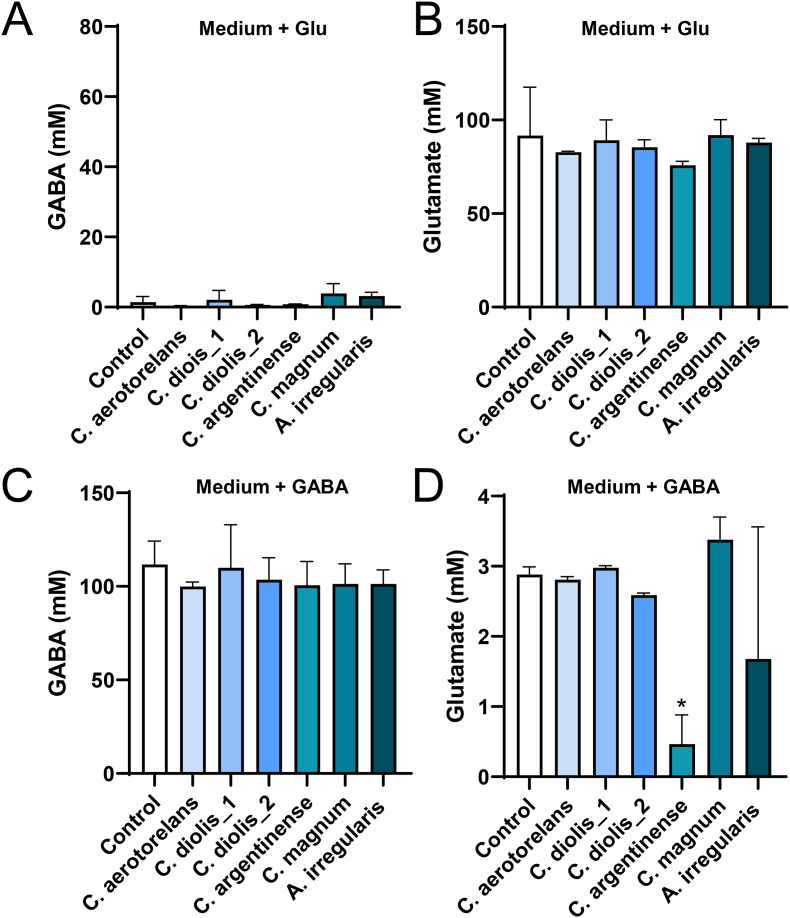
**GABA production and consumption ability of isolated Clostridiales.** Isolated Clostridiales strains were cultured in media supplemented with glutamate (A, B) or GABA (C, D) to determine the changes in GABA (A, C) and glutamate (B, D) concentrations in the culture supernatants. Data are presented as mean ± SD of two independent experiments. *P < 0.05 vs. control without bacteria (one-way ANOVA with Dunnett's test).

The isolated strains were then evaluated for their ability to produce or consume GABA. The bacterial isolates were cultured in media supplemented with glutamate or GABA to determine the changes in GABA and glutamate concentrations in the culture supernatants. *L. brevis* NBRC 12005, a well-known GABA producer, was used as the control. The *E. gallinarum* strain consumed large amounts of glutamate and produced high levels of GABA when cultured in medium supplemented with glutamate (Fig. 1A and B). The *E. gallinarum* strain converted more glutamate to GABA than the *L*. *brevis* strain, suggesting that *E. gallinarum* is one of the primary GABA producers in the intestine. In contrast, the *E. gallinarum* strain showed no GABA consumption ability as the GABA concentration in the culture supernatant did not change when cultured in a medium supplemented with GABA (Fig. 1C). The *E. gallinarum* strain reduced glutamate in the GABA-supplemented medium, also indicating that the strain converted glutamate to GABA, even though the original glutamate concentration in the medium was too low to significantly increase the GABA concentration in the high-GABA-concentration medium (Fig. 1D). The GABA and glutamate concentrations in the culture supernatants of all six Clostridiales strains did not change, except that *C. argentinense* reduced the glutamate concentration in the GABA-supplemented medium, indicating that none of these strains can produce or consume GABA (Fig. 2A–D). The *C. argentinense* strain may consume glutamate, which is converted to metabolites other than GABA.

### 3.3 Analysis of gad cluster of *E. gallinarum* strain

**Fig. 3:**
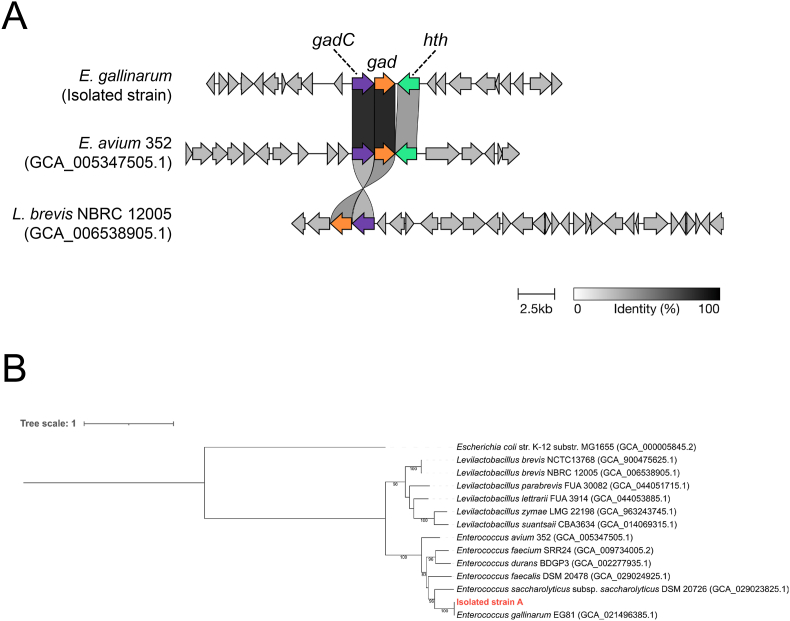
**Characterization of *gad* cluster of *E. gallinarum* strain.** (A) Comparison of *gad* clusters among the *E. gallinarum* strain isolated in this study, *E. avium* 352, and *L. brevis* NBRC12005. *hth*, gene encoding helix–turn–helix domain-containing protein. (B) Phylogenetic tree based on *Rad51* sequences of seven *Enterococcus* strains including the isolated strain and *E. gallinarum* EG81 with a reported complete genome sequence, six *Levilactobacillus* strains, and an *Escherichia coli* strain.

We next studied the presence of the *gad* gene in the genome of the *E. gallinarum* strain, which was found to produce GABA from glutamate. The genomic data assembled by next-generation sequencing was biologically annotated using DFAST [23]. As shown in Fig. 3A, the isolated *E. gallinarum* strain possessed a *gad* gene that formed a cluster together with *gadC*, which encodes a glutamate-GABA antiporter, and a gene encoding a helix-turn-helix domain-containing protein. Among the bacteria belonging to the genus *Enterococcus*, the presence of the *gad* gene has been reported in *E. avium* strains G-15 [28] and SDMCC050406 [29]. The *gad* gene clusters were therefore extracted from the genomic sequences of *E. avium* strain 352 (GCA_005347505.1) and *L. brevis* NBRC12005 (GCA_006538905.1) and compared with the isolated *E. gallinarum* strain (Fig. 3A). The *gad* cluster was similar among these three strains, with the *gadC* gene aligned upstream of *gad*. The homology of the *gad* and *gadC* sequences of the isolated *E. gallinarum* strain was higher with those of *E. avium* than with those of *L. brevis*. Notably, the amino acid sequences of these gene products were more than 85% homologous between the *E. gallinarum* and *E. avium* strains. This is consistent with the phylogenetic tree based on *Rad51* sequences (Fig. 3B).

Collectively, these data confirm that *E. gallinarum* can produce large amounts of GABA by decarboxylating glutamate.

## 4 Discussion

The roles of gut microbiota in maintaining the mental health of the host via the gut-brain axis have been recognized. Some intestinal bacteria produce GABA from glutamate, suggesting a role of intestinal GABA derived from gut microbiota in the gut–brain axis. We previously showed that the intestinal GABA concentration depended on the composition of gut microbiota and affected the behavioral characteristics of the host, such as anxiety behaviors [20]. In this study, we found that an *E. gallinarum* strain isolated from murine intestine produced high levels of GABA. Some bacterial strains belonging to *Enterococcus* such as *E. avium* JS-N6B4, *E. faecium* BS5, and *E*. *malodoratus* SJC25 produce GABA [[30], [31], [32]]; however, the GABA-producing genes of these strains have been scarcely analyzed. Only a few studies have identified the *gad* gene and its cluster structure in *E. avium* [28,29]. Furthermore, most of the *Enterococcus* strains used in these analyses were isolated from food sources. The GABA production capacity of *Enterococcus* colonizing the intestine, as well as the genes involved in GABA production, remain largely uncharacterized. There are few reports on GABA production by *E. gallinarum*, and we found no reports indicating the presence of the *gad* gene in this species. This is the first report to isolate a gut-resident *E. gallinarum* strain with a high GABA-producing ability and clarify the presence and structure of its *gad* gene cluster. However, further functional validation is required to characterize this strain by elucidating the regulatory mechanisms of GABA production and its impact on host physiology. Although the isolated strain may be a primary GABA producer in the intestine, other strains may also produce GABA because we obtained a small number of strains belonging to Lactobacillales in our study.

*E. gallinarum* is a Gram-positive coccus typically found in the intestines of humans, mice, and other animals. It is non-pathogenic in healthy individuals but opportunistically pathogenic in immunocompromised individuals. *E. gallinarum* is naturally resident to vancomycin because of the presence of *vanC*. This differs from vancomycin-resistant (VRE) with acquired resistance, which are often problematic. Some *Enterococcus* strains produce antimicrobial peptides called bacteriocins that inhibit colonization by VRE. Therefore, *Enterococcus* strains that are less pathogenic and have beneficial effects are expected to be useful as probiotics. In fact, many *Enterococcus* strains, including those belonging to *E. gallinarum*, are effective in humans and livestock [[33], [34], [35]], and some are used commercially.

The *gad* cluster structure of *E. gallinarum* was similar to that of *E. avium* and *L. brevis*. All of these possessed the *gadC* gene upstream of *gad* (Fig. 3A). The *gadC* gene encodes an antiporter that transports GABA produced in cells to the extracellular space via exchanging GABA with glutamate. The conversion of glutamate to GABA consumes protons in bacterial cells. Additionally, the exchange of GABA and glutamate mediated by the *gadC* product essentially involves the release of intracellular protons into the extracellular space, enabling bacteria to adapt to acidic environments. The *E. gallinarum* strain isolated in this study, as well as most GABA-producing bacteria, likely possesses this type of gene cluster with *gadC* to maintain intracellular pH under acidic conditions.

The *gadR* gene, which encodes a transcriptional regulator, is often found within the *gad* cluster in the Lactobacillaceae family [36]. The *gadR* product activates the transcription of *gad* and *gadC* within the cluster as well as other genes related to the GAD system. *gadR* is upstream of *gadC* in both *L. brevis* NBRC12005 used in our experiment and *L. brevis* NCTC13768 in the phylogenetic tree in Fig. 3B. The amino acid sequence encoded by *gadR* is 100% identical between these strains. *E. avium* strain 352 possesses a gene with the same function, but this gene product has low sequence homology (26.14%) with that of *L. brevis*. The isolated *E. gallinarum* strain also had a candidate gene, but the gene was located far from the *gad* cluster, suggesting that *gadR*-mediated regulation differs among the bacterial species. In addition, a gene encoding a hypothetical protein with a helix–turn–helix domain, often found in transcription factors, was located downstream of *gad* in the *E. gallinarum* strain (Fig. 3A). This gene product shared 48.43% sequence homology with that of *gadR2,* which is located downstream of *gad* and encodes another transcriptional activator in *E. avium* G-15 [28]. These observations suggest that the core cluster composed of *gadC* and *gad* is universally essential; however, the *gadR* system varies among bacterial species. Variations in the sequences and genomic loci of these transcriptional regulators may influence the efficiency of GABA production; however, further experimental validation, such as gene knockout studies, will be required. Consequently, the *E. gallinarum* strain may produce higher levels of GABA than the *L. brevis* strain. Given that the effects of probiotics are often strain-specific, it is crucial to compare and characterize *gad* gene clusters across a wide range of strains.

Limitations of this study include the lack of a detailed investigation into the mechanisms regulating GABA production, an evaluation regarding the actual GABA contribution of this strain within the complex intestinal environment, and an analysis of its impact on host physiology. Further studies are required to clarify the relationship between the abundance of *E. gallinarum* and intestinal GABA concentrations or diseases. These studies, together with research on survival in the gastrointestinal tract and safety, will enable the evaluation of this strain's usefulness as a probiotic.

## CRediT authorship contribution statement

**Mion Ikegami:** Formal analysis, Investigation, Writing – original draft. **Miyu Yamashita:** Formal analysis, Investigation, Writing – review & editing. **Ren Kadowaki:** Formal analysis, Visualization, Writing – review & editing. **Gaku Harata:** Conceptualization, Formal analysis, Investigation, Writing – review & editing. **Kenji Miyazawa:** Supervision, Writing – review & editing. **Yusuke Nakanishi:** Supervision, Writing – review & editing. **Kyoko Takahashi:** Conceptualization, Funding acquisition, Investigation, Supervision, Writing – original draft, Writing – review & editing.

## Declaration of competing interest

RK, GH, and KM are employees of Takanashi Milk Products Co., Ltd. (Kanagawa, Japan). The remaining authors have no conflicts of interest.

## Data Availability

Data will be made available on request.
